# Supplementary material for: Clinical Manifestation, Management, and Outcomes in Patients with COVID-19 Vaccine-Induced Acute Encephalitis: Two Case Reports and a Literature Review
Source: Vaccines (Basel). 2022 Jul 31;10(8):1230. doi: 10.3390/vaccines10081230 (PMC9414071; doi:10.3390/vaccines10081230)
Supplement: Supplementary file 1 [file vaccines-10-01230-s001.zip › vaccines-1847691-supplementary.pdf]

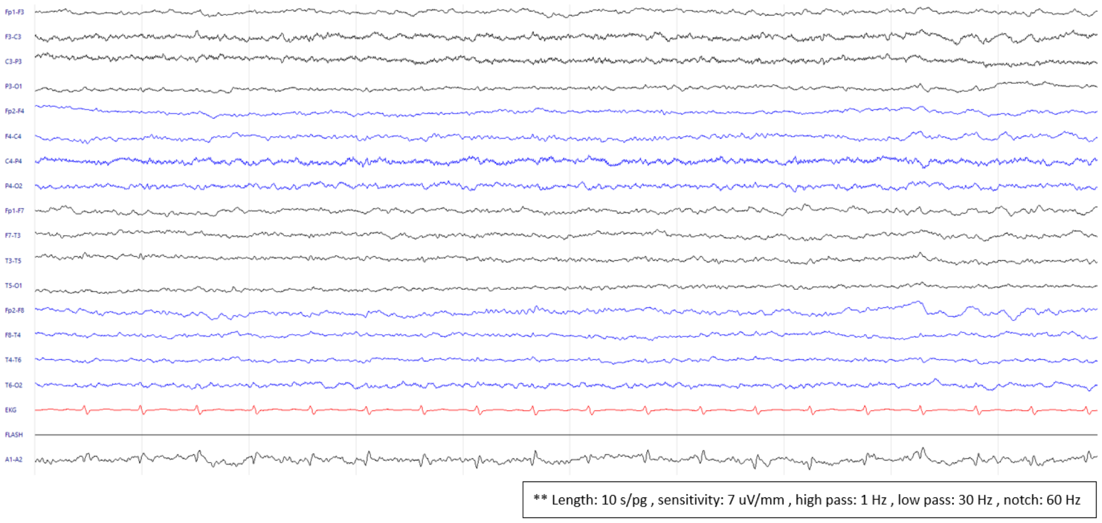

Supplementary Figure S1. Electroencephalogram demonstrated a continuous diffuse slowing in the theta and delta ranges (Case 2).

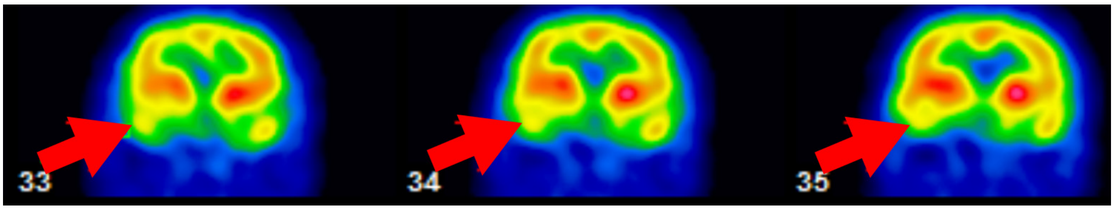

Supplementary Figure S2. The cerebral perfusion scan with single-photon emission computed tomography (SPECT) that indicated hypoperfusion in the right temporal region (Case 2).
